# Genome Sequence of *Halomonas* sp. Strain A3H3, Isolated from Arsenic-Rich Marine Sediments

**DOI:** 10.1128/genomeA.00819-13

**Published:** 2013-10-10

**Authors:** Sandrine Koechler, Frédéric Plewniak, Valérie Barbe, Fabienne Battaglia-Brunet, Bernard Jost, Catherine Joulian, Muriel Philipps, Serge Vicaire, Stéphanie Vincent, Tao Ye, Philippe N. Bertin

**Affiliations:** UMR7156 Université de Strasbourg/CNRS, Génétique Moléculaire, Génomique, Microbiologie, Département Micro-organismes, Genome, Environnement, Strasbourg, Francea; Laboratoire de Finition, CEA-IG-Génoscope, Évry, Franceb; BRGM, Orléans, Francec; Plateforme Biopuces et Séquençage, IGBMC, Illkirch, Franced

## Abstract

We report the genome sequence of *Halomonas* sp. strain A3H3, a bacterium with a high tolerance to arsenite, isolated from multicontaminated sediments of the l’Estaque harbor in Marseille, France. The genome is composed of a 5,489,893-bp chromosome and a 157,085-bp plasmid.

## GENOME ANNOUNCEMENT

*Halomonas* sp. strain A3H3, a slightly halophilic bacterium (0.5 to 9% NaCl) (1), was isolated from the harbor sediments of l’Estaque in the south of France. These sediments are highly contaminated with various metals, metalloids, and organic compounds, particularly with arsenic (653 µg/liter in sediment interstitial water and 165 mg/kg in solid phase) (2, 3). A 16S rRNA-based phylogenetic analysis revealed close similarity to *Halomonas neptuniae* strain Eplume1 (GenBank accession number NR_027218.1).

The genome of *Halomonas* sp. A3H3 was sequenced from one paired-end and one 500-bp mate-paired library of 5-kb inserts. The resulting reads were assembled into 131 contigs using SOAPdenovo and GapCloser assemblers, achieving 150-fold coverage. The sequence assembly completed by optical mapping (4) resulted in two scaffolds representing a total of 5,646,978 bp. The genome consists of a 5,489,893-bp chromosome with a 54.59% average GC content and 5,143 coding sequences (CDS) and a 157,085-bp plasmid with a 61.97% average GC content, 221 CDS, and a coding density of 89.38%. The size of the plasmid was experimentally validated using Wheatcroft’s method (5). A total of 5,568 CDS, 48 tRNAs, 13 miscellaneous RNAs, and 4 rRNA genes were predicted and annotated using the MicroScope platform (6). A total of 31.55% of CDS are of unknown function.

Best synteny and gene conservation were found with *Halomonas* sp. strain HAL1 (7), *Halomonas boliviensis* LC1 (8), and *Halomonas* sp. strain TD01 (9) genome sequences, with 64.7, 63.7, and 58.9% of genes in syntons, respectively. Remarkably, the genome of *Halomonas* sp. A3H3 is about 1.5 Mb larger, suggesting the acquisition of functions allowing better adaptation to its environment, e.g., genes coding for tripartite ATP-independent periplasmic (TRAP) transporters for substrate uptake (10) or salicylate degradation. In contrast, no *car* or *bph* operon was identified, although *Halomonas* sp. A3H3 uses aromatic compounds such as biphenyl and carbazole as sole carbon sources, which suggests the possible use of alternative degradation pathways. The presence of numerous transposases or phage-related CDS throughout the genome sustains a large number of rearrangements.

*Halomonas* sp. A3H3 tolerates up to 29 mM As(III) and more than 106 mM As(V). Four *ars* operons (two on the chromosome and two on the plasmid) containing *arsD*, *arsA*, *acr3*, *arsC, arsH*, and/or *arsR* genes (11, 12, 13) were identified. Importantly, *aioBA* genes (14) located on the plasmid may also have been acquired by horizontal transfer. The bacterium oxidizes 100 mg/liter arsenite under aerobic conditions or under anaerobic conditions in the presence of nitrate used as an alternative final electron acceptor, as supported by the presence on the chromosome of the *narGHJI* operon (15). *Halomonas* sp. A3H3 is motile by means of peritrichous flagella, the genes of which are clustered in a large chromosomal region. The motility of the bacterium increases with arsenite, as shown by swarming assays, which further supports the use of arsenite oxidation in the energy metabolism of *Halomonas* sp. A3H3.

Further studies will provide insights into the strategies evolved by *Halomonas* sp. A3H3 to deal with the toxic compounds present in marine anaerobic harbor sediments.

### Nucleotide sequence accession numbers.

The whole-genome sequence has been deposited at DDBL/EMBL/Genbank under the following accession numbers: contigs, CBRE010000001 through CBRE010000131; scaffolds, HG423310 through HG423342; and chromosomes, HG423343 and HG423344.
